# The Promotion of Data Sharing in Pharmacoepidemiology

**DOI:** 10.1163/15718093-12341323

**Published:** 2014-06

**Authors:** Nayha Sethi

**Affiliations:** Research Fellow, School of Law, University of Edinburgh, UK

**Keywords:** pharmacoepidemiology, secondary uses, data reuse, health research, data protection, data sharing

## Abstract

This article addresses the role of pharmacoepidemiology in patient safety and the crucial role of data sharing in ensuring that such activities occur. Against the backdrop of proposed reforms of European data protection legislation, it considers whether the current legislative landscape adequately facilitates this essential data sharing. It is argued that rather than maximising and promoting the benefits of such activities by facilitating data sharing, current and proposed legislative landscapes hamper these vital activities. The article posits that current and proposed data protection approaches to pharmacoepidemiology — and more broadly, re-uses of data — should be reoriented towards enabling these important safety enhancing activities. Two potential solutions are offered: 1) a dedicated working party on data reuse for health research and 2) the introduction of new, dedicated legislation.

## 1 Introduction

This article posits that a responsive, adaptable and flexible approach to data reuse for research is imperative for patient safety, and uses pharmacoepidemiological research as a case-study to do so. Pharmacoepidemiological research provides an important case-study because it straddles the divide between traditional monitoring of patient safety, within pharmacovigilance and wider reuse of health data in the research setting, which also contributes significantly towards ensuring patient safety.

It is argued that current categorisations within the law of different conditions of health data reuse present an artificial distinction between such interrelated activities. The article begins by considering pharmacoepidemiology and the numerous benefits of the discipline in terms of enhancing patient safety. The crucial importance of patient data to these activities is highlighted. Self-evidently, the more accurate, complete and readily available these data are, the clearer the picture about medicine safety and efficacy becomes. Data protection legislation and the related obligations to which it gives rise play a fundamental role in determining whether access to data is granted and under which circumstances. Given the reliance on data and the proposed legislative changes to data protection legislation which are currently under consideration within the European Union, it is timely to consider whether the current legislative landscape adequately facilitates pharmacoepidemiological activities and whether proposed changes will also do so, or change the dynamic for ill.

It is argued here that rather than maximising the benefits of such activities by enabling sharing, the current and proposed legislative landscapes are unnecessarily inhibitive. This manifestly does not reflect the public interest in scientifically sound medical research and in ensuring patient safety. Nor does it appreciate the obstructive effects which arbitrary categorisation of data sharing activities can have. It is proposed here that the law can be framed so as to act as an enabler, and a cultural driver for sharing in appropriate circumstances. This does not denigrate the important role of the law in inhibiting inappropriate data sharing, however a balance must be achieved, rather than the current status quo where data reuse is often prevented even in ethically and scientifically sound projects and despite the fact that patient safety stands to benefit. Responsive, adaptive and operationalisable solutions are required. Two options for improving the situation are briefly outlined: the first comes in the form of a dedicated working party tasked with considering issues arising from reuse of health data for research; the second is a proposal for new legislative provision that specifically addresses data reuse for research.

First, a preliminary note; reference is often made within the health research literature to ‘secondary uses of data’ this is to say, data ‘initially collected for another purpose’.^1^ While this might seem to be simply a matter of semantics, it is argued here that reference to the term ‘secondary uses’, is unhelpful and antithetical to one of the key messages in this article, viz, that such data reuses are valuable and merit due recognition and, when appropriate, prioritisation. The language that we use is all-important because the term ‘secondary use’ tends to imply ‘inferior’ use. A more helpful framing might be achieved via rephrasing ‘secondary uses’ of data to ‘data reuse’. Accordingly, the term ‘data reuse’ is used throughout this article.

## 2 Pharmacoepidemiology: An Overview

This initial section considers the crucial role which pharmacoepidemiology plays in patient safety and why data access is key in facilitating such activities. First, however, a brief summary of what pharmacoepidemiology entails, and how it has developed since its emergence is offered.

### 2.1 What Is Pharmacoepidemiology?

Pharmacoepidemiology is ‘the study of the use, and effects, of drugs and other medical devices in large numbers of people’,^2^ ‘it aims to examine all detectable effects, whether beneficial or adverse’.^3^ It encompasses many activities including the study of adverse drug reactions (ADRS). An adverse drug reaction is described as:

an unwanted or harmful reaction experienced following the administration of a drug or combination of drugs under normal conditions of use, which is suspected to be related to the drug. The reaction may be a known side effect of the drug or it may be new and previously unrecognised.^4^

Pharmacoepidemiology is considered for present purposes, as the study of the use of effects of drugs amongst large populations after a drug has received its licence, in the post-marketing phase.^5^

Few laws existed to regulate drug marketing and development at the time of the Thalidomide revelations^6,7^ and a clear need for addressing drug safety issues was identified, guidelines were developed across different countries in order to monitor drugs for ADRS. Examples include: the US Kefauver-Harris Amendments^8^ the UK Yellow Card Scheme^9^ and Directive 65/65/EEC to safeguard public health concerning production and distribution of medicinal products.^10^ Subsequently, a plethora of legislation has emerged,^11^ rendering patient safety a core aspect of drug development and research. Numerous national level pharmacoepidemiology centres and coordinated research initiatives exist.^12,13,14^ Whilst drug surveillance has significantly enhanced patient safety, the prevalence and under-reporting of ADRS remains.^15,16^

### 2.2 Why Is Pharmacoepidemiology so Important for Patient Safety?

Pharmacoepidemiological studies are integral to patient safety, when a drug is available to the wider population and throughout the lifetime of each patient who consumes the medication. Studies affect other patients with similar illnesses by influencing the availability of such drugs and the ability to make informed decisions about drug suitability given knowledge about patient experiences. Without pharmacoepidemiology, our understanding of the safety and efficacy of medicines is limited; such studies offer a much more complete picture about drugs and allow us to detect risks which would only appear when a large number of individuals have been exposed to a medicine. Clinical trials typically exclude or have limited involvement of certain population groups^17,18,19,20^ rendering extrapolation of results to these groups in the population at large particularly difficult.^21,22^ Clinical trials traditionally occur over limited time periods. Pharmacoepidemiology enables us to monitor the efficiency, effects and risks of a drug in the longer-term.^23^ We can also learn how drugs react with each other in patients with co-morbidities (also typically and routinely excluded from clinical trials).^24^ Pharmacoepidemiological studies can lead to withdrawal of a drug from the market and changes to the product label, indications for use and terms of availability.^25^ Additionally, such studies reveal unexpected benefits leading to use for treatment of diseases other than those for which the drug was originally developed, i.e., serendipitous drug discovery.^26,27,28^

## 3 Data as a Key Component in Patient Safety

More accurate, accessible, and complete data offer more complete drug profiles which increase patient safety and facilitate the benefits of cost efficiency. Data sharing also plays a key role to ‘accelerate health improvements’ more generally.^29^ To fully appreciate the critical role which data play in enabling such activities, an overview of some methods and sources of data which pharmacoepidemiological studies rely upon is helpful.

### 3.1 Spontaneous Reporting (SR)

Spontaneous Reporting(SR) is a flawed but essential tool used to flag ADRS.^30^ In the UK, doctors are obliged to report ADRS^31^ and patients can report them via the Yellow Card Scheme,^32^ particularly important as prescribing for children can often be off-label.^33^ Under-reporting of ADRS is problematic,^34,35^ due to failure to associate the ADR with the new drug^36^ or to understand when an ADR should be reported.^37^ This can be a challenge across the EU.^38,39,40^ Whilst all ADRS must be reported for black triangle status drugs, for established drugs, only serious ADRS must be reported, leaving a significant gap in knowledge about more minor ADRS.^41,42^ SR ‘offers low evidence of risks associated with medicines’ but the ‘highest levels of uncertainty regarding causality’.^43,44,45^ If we are to achieve a reliable evidence base for serious and minor ADRS, and offer more certainty than that a drug is ‘apparently safe based on partial information’,^46^ then we must go well beyond SR.

### 3.2 Data Linkage (DL)

One method employed in pharmacoepidemiology, particularly significant here, is data linkage, which ‘…brings together information from two or more records from independent sources that are perceived to belong to the same individual, family, event or place’.^47^ DL relies upon reuse and linking of data sources such as: electronic health records; routinely collected administrative data such as prescribing data, hospital admissions, mortality and morbidity data, cancer registries etc.^48^ Reusing data has many strengths, including that data are ‘collected prospectively without knowing the research question issues of bias.’^49^ DL provides a cost-effective means of analysing pre-collected data and wielding results that may not be possible or ethically or economically viable in other settings such as clinical trials.^50^ Such studies can involve linking several different datasets. Numerous national and European initiatives are dedicated to maximising the research potential of reusing data for health research.^51^ Database studies are conducted throughout North America and Europe.^52,53,54^

The major funding councils in the UK have collaborated on a £19 million funding call reflecting their appreciation of the importance of ‘the health research potential offered by linking electronic health records with other forms of routinely collected data and research datasets’.^55^ Indeed, the new UK-network of health linkage centres — the ‘Farr Institute’ — has been established to ‘deliver high-quality, cutting-edge research linking electronic health data with other forms of research and routinely collected data’.^56^ Through data linkage, pharmacoepidemiological activities straddle questions of patient safety both immediately after a drug has been licensed, but also in the long-term, where studies involve linkage of numerous data sets on large populations over significant time periods. Whilst major research funders appear to be moving in the direction of promoting and prioritising such research, the legislative regime(s) in this area are lagging behind, hampering these crucial activities, let us consider how.

## 4 Data Governance Regime: Losing Sight of Priorities

Although data linkage brings with it many advantages, including access to data which are already collected and which can be traced over long periods of time, reuse of data for data linkage purposes faces significant regulatory hurdles. This next section considers the regulatory hurdles currently inhibiting studies aimed at improving patient safety. Detailed accounts laying out the legislative landscape governing reuse of health data are recounted elsewhere.^57^ A summary of the problems with the current landscape as a whole sets the scene.

### 4.1 Current Governance Regime

The key message from literature discussing governance of reuse of health data is clear: the current landscape is complex and unduly burdensome.^58^ That the European Data Protection Directive (EuDPD) is open to interpretation by Member States (of the EU) perpetuates divergent approaches to data sharing across the European Union.^59^ The disproportionate procedures which researchers must navigate to access data are also well documented.^60^ A particular issue is the myriad, often repetitive and time-consuming approvals procedures which must be fulfilled prior to data access.^61^

The point is not that approvals procedures are unnecessary. On the contrary, ensuring that robust and proportionate legal and ethical standards are met both prior to and during the use of sensitive data is non-negotiable. What is crucial is that any standards which must be met — including data access and approval procedures — are appropriate to the level of perceived risk associated with a study. Equally, such standards should reflect the important goal of facilitating important health research which can contribute to patient safety, in line with the fundamental principle of ensuring the free flow of data — as provisioned in the EuDPD. It is paramount that legislation is not only operationalisable on a practical level, but that it enables data sharing in appropriate contexts, rather than prohibiting sharing, especially when prohibitions are based on artificial and impractical distinctions made in law.

Let us consider, the dominant ‘consent or anonymise’ approach to current data reuse for health research in the UK.^62^ This is understood as implying that where patient consent cannot be obtained for using data for a research project, then the data should be anonymised by default. Obtaining consent is not always possible or practical, particularly when one considers:

…the cost of contacting hundreds of thousands, or even millions, of individuals. Or trying to reach them years after their health care encounter, when many may have relocated, some may have died, and some may not want to be reminded about an unpleasant of traumatic experience.^63^

Notwithstanding, and although anonymisation of data can facilitate research, the process can also be problematic. It is said, for example, that complete anonymisation or de-identification^64^ of data is in fact a myth,^65^ if not ‘very difficult to achieve in practice’.^66^ Moreover, ‘…the security of personal records in databases cannot be guaranteed through anonymisation procedures’.^67^ This being said, anonymisation can still considerably diminish the likelihood of re-identification where this is the desired goal.^68^ Rather than discussing the impossibility of re-identification of individuals, however, a more helpful and realistic framing of the discussion might be around *likelihood*^69^ of re-identification as opposed to judgements being made around *possibility* of re-identification; if we accept that anonymisation is a myth, then re-identification would always be possible, this offers a perfect example of an impractical categorisation within the legislative landscape.

Indeed, eradicating the possibility of re-identification is not always the desired practical goal; an important aspect of pharmacoepidemiological and more general^70^ data linkage research is traceability of data. This enables longitudinal studies which track the effects of medication over a patient’s lifetime, drug safety issues may only arise after: a period of time, the development of new illnesses and/or the introduction of new medications to the patient. Re-identification of patients may be necessary ‘when clinically relevant information arises during the course of a study which might have a direct impact on the treatment of a patient. In such cases, ethical principles demand recontacting and informing all relevant patients about the findings’.^71^ Further, traceability facilitates follow up studies of cohorts. Traceability implies that the patients are either identifiable, or that their data are pseudonymised, so that re-identification is possible.

Pseudonymisation is one mechanism which does enable traceability whilst alleviating some concerns around using identifiable data. This is ‘the process of distinguishing individuals in a dataset by using a unique identifier which does not reveal their ‘real world’ identity.’^72^ It is considered a ‘powerful and flexible tool for privacy protection in databases’. It allows ‘linking data associated with the true individual, through the pseudo-IDS, irrespective of the collection time and place’.^73^ Pseudonymisation is one of several mechanisms which speak to current flexibilities around conducting data linkage^74^ however, even here, challenges exist. The extent to which pseudonymisation is (in)effective will depend upon the techniques used and the specific contexts of the research/data use.^75^ The method may ‘not be adequate for many research purposes’.^76^

Two important points can be made here. First, the lines between anonymous, pseudonymous and personal data are not clear cut. Current legislation unhelpfully assumes that these distinctions can be made easily, which is not the practical reality. Further, it is argued that we cannot and should not simply rely on technical processes to remedy the problems plaguing reuses of health data. Indeed, reliance on technical processes in turn gives rise to confusion around definitions and around which techniques are acceptable to satisfy thresholds of ‘anonymisation’, ‘pseudonymisation’ and relatedly, to what counts as ‘personal data’.^77^ We need to consider the purposes, contexts^78^ and value of reusing health data. It has been suggested that the confusing landscape governing data reuse, which makes difficult demands on researchers and data custodians in negotiating with ill-defined terms, has led to a culture of caution, with fear of sanctions for data sharing which may actually not be inappropriate.^79^ The focus here is on the effects of data protection legislation on conducting pharmacoepidemiological — and more broadly — health research through data reuse. However, it is also important to acknowledge that cultural attitudes and practices towards data reuse must play an undeniable role in how data are used,^80^ but equally, the law plays a significant role in shaping the regulatory landscape. Consequently, a clearer, flexible and practicable framing of data reuse is required.

Another solution which does accommodate consideration of the purposes of reuse involves taking principled proportionate approaches to governance, as I have argued elsewhere.^81^ One example of taking such an approach is via authorisation, whereby a proxy decision-maker in the form of an individual or body can authorise a linkage. However, pre-existing mechanisms within the current landscape which facilitate data linkage are limited. Further, the flexibility currently offered by pseudonymisation, which facilitates many significant studies based on reuse of health data is put at risk in the proposed Data Protection Regulation.^82^

### 4.2 Proposed Legislative Reform: What Is Around The Corner?

We have briefly considered above the current regulatory landscape governing the reuse of data for research, concluding that a regulatory approach which better promotes sharing data in appropriate circumstances is needed. The EuDPD was drafted at a time when the use of the Internet to share and collate data was in a nascent stage.^83^ Internet use has proliferated, alongside globalisation, technical advances and the numerous internet-related privacy scandals in news headlines. Issues of transfer of data to countries outside the EU have also emerged.^84^ It has become clear that the current Directive is no longer fit for purpose.^85^ Legislative reform is underway and rather than adopting another directive, a regulation has been proposed to replace the ‘patchwork of national laws with a single set of rules’.^86^ A robust analysis of the proposed legislation is not offered here, given that full agreement has not yet been reached on the final text. Rather, this section considers which direction the current text (which has just been approved by the European Parliament) might steer data reuse towards, concluding that if the European Council approves the current text, the end point for such research could be even worse than the current unacceptable situation.

The initial draft proposal of the General Data Protection Regulation was advanced by the European Commission in January 2012.^87^ Two years and several delays later, it has still not been adopted; unsurprising when we consider the myriad stakeholder concerns involved. The drafters have been charged with the unenviable task of legislating on a wide range of data protection issues. Whilst the proposed Regulation could help to clarify issues around the processing of personal data for the purposes of monitoring drug safety pre-marketing,^88^ it is argued here that an opportunity is being missed in improving how data reuse in post-marketing pharmacoepidemiological studies and wider health research — also integral for patient safety — can take place. The lead Committee on Civil Liberties, Justice and Home Affairs (LIBE) voted to adopt recommendations from Rapporteur Albrecht (the Albrecht Report)^89^ which proposed numerous amendments to the Regulation. Certain amendments, which were proposed in January 2013, have raised significant concerns amongst the research community.^90,91^

The European Parliament has approved the amended text which appears to be missing out on the opportunity of realigning the due regard for one of the fundamental principles upon which the EuDPD was originally founded; the free flow of data which corresponds to promoting sharing data in appropriate circumstances. The draft Regulation includes clear acknowledgement that:

The centrepiece of existing EU legislation on personal data protection, Directive 95/46/EC3, was adopted in 1995 with two objectives in mind: to protect the fundamental right to data protection and to guarantee the free flow of personal data between Member States.^92^

Further, ‘[t]he current framework remains sound as far as its objectives and principles are concerned’,^93^ references to achieving the dual objectives of data protection and ensuring the free flow of data are made throughout the draft Regulation. Indeed, the very wording of the Regulation title is ‘Regulation of the European Parliament and of the Council on the protection of individuals with regard to the processing of personal data and on *the free movement of such data*’.^94^ It is argued here that despite such explicit articulations of the dual goals of protecting privacy and promoting free movement of data, the clearly important role of protecting individual privacy rights distracts from the equally important goal of facilitating data sharing, in this case, facilitating the reuse of data for patient safety and health research. Let us consider in more detail the relevant provisions and amendments to the draft regulation, (which emanated from the Albrecht Report) to demonstrate how this is so.

The Albrecht Report proposed (and Parliament agreed) that Article 81 of the original draft Regulation be revised to read as follows:

Processing of personal data concerning health which is necessary for historical, statistical or scientific research purposes, shall be permitted *only with the consent of the data subject*,^95^ *and shall be* subject to the conditions and safeguards referred to in Article 83.^96^

It is not clear to this author precisely where pharmacoepidemiological studies which aim to investigate long-term post-marketing effects of drugs fits in the legislation. If such studies are viewed as fitting under ‘scientific research purposes’ as many data linkage studies are, then the implications of this amendment would be that research using identifiable data where consent has not been obtained will be prevented.

Article 7 of the Albrecht Report requires consent to be “specific, informed and explicit”. The (im)practicalities around obtaining consent^97^ and the over-reliance of consent as a panacea to all potential privacy concerns have been discussed at length elsewhere,^98^ but clear practical hurdles include resources such as time and budget to facilitate obtaining consent, if even possible. These barriers could prevent many studies going ahead.^99^

With regards to pseudonymisation, we considered above that this process provides at least some flexibility within the current landscape however there is a lack of sufficient clarity with regards to whether pseudonymised data will fall under the scope of ‘personal data.’^100^ This being said, a recent Art 29 DPWP Opinion on anonymisation has raised the bar with regards to anonymisation and suggests that pseudonymised data ‘stays inside the scope of the legal regime of data protection’.^101^ If the new regulation text is approved by the European Council, then “[t]he inclusion of pseudonymised data within the scope of ‘personal data’ could dramatically increase the regulatory burden on research.”^102^ This will place even tighter restrictions upon the extent to which these data can be reused. The UK Information Commissioner’s Office noted that if personal data is to be defined so as to include pseudonymised data, then ‘…it is important to be clear that a wide definition plus all the associated rules in full would not work in practice.’^103^ Another Albrecht amendment proposed that pseudonymised health data could be used without consent but only if the threshold of ‘exceptionally high public interest’ is met.^104^ Indeed, under Article 42 of the original draft Regulation: ‘historical, statistical and scientific research purposes’ were included as legitimate reasons for ‘derogating from the prohibition of processing sensitive categories of data’.^105^ The drafters of the Albrecht Report state:

Processing of sensitive data for historical, statistical and scientific research purposes is not as urgent or compelling as public health or social protection. Consequently, there is no need to introduce an exception which would put them on the same level as the other listed justifications.^106^

The clear value statement included in the quote above is problematic given the significant contributions pharmacoepidemiological studies make towards patient safety. Again, exactly where such research sits with regards to ‘scientific research’ or ‘public health’ is questionable and it is argued that the distinction, based on ‘urgency’ between the different uses of data is unhelpful and can lead to prohibiting important activities.

Thus far, we have considered why availability of health data is crucial for patient safety, as guaranteed by pharmacoepidemiological research. We have outlined some reasons why the current data protection regime is sub-optimal with regards to facilitating such activities. Key points which have emerged are that researchers can use different technical methods for privacy protection but none is free from issues: particularly given the fact that the more information which is joined together from different sources, the more the likelihood of identifiability will increase,^107^ as with ‘jigsaw’ techniques.^108^ Further, there is uncertainty and variance in practice around pseudonymisation techniques.^109^ Such observations serve as examples of how unhelpful it can be to focus solely on categorizing types of data and types of use when the ultimate goal of such studies is to ensure patient safety. Pharmacoepidemiology as we have considered, speaks to patient safety at different levels, not only in the context of pre-marketing surveillance of drug reactions, but also in the wider context of health research studies which focus on drug effects in wide populations over a sustained period of time, but nonetheless contribute significantly towards patient safety. We have briefly considered above the effects which the amended draft regulation might have on data reuse in health research if the European Council follows the Parliament in approving the text.

It has been considered that proposed changes to the European framework may be particularly detrimental to realising the potential of data reuse in health research, especially if pharmacoepidemiological studies fit under ‘scientific research purposes’, if pseudonymisation is brought to fit under the scope of personal data and if explicit consent is required prior to data reuse for such data.

## 5 Reorienting the Research Path

This final section suggests that a reorientation of how the reuse of data is prioritised within data protection legislation could improve the current situation. A role is envisaged for the law as an enabler of data sharing, where this is for scientifically and ethically robust reuse of patient data not only for pharmacoepidemiological research, but for wider health research. Two means of enacting this role are posited here: the first is in the form of the establishment of a dedicated Working Party on data reuse and the second in the form of bespoke legislation specifically aimed at data reuse for research. An overview of each is offered alongside reasons why these options might be considered.

### 5.1 The Establishment of a Dedicated Working Party on Data Reuse

Issues relating to reuse of health data for research are currently considered at an EU level by the Article 29 Data Protection Working Party (Art 29 DPWP). The Party was established in 1996 alongside the EuDPD. The Art 29 DPWP comprises of: ‘a representative of the supervisory authority (ies) designated by each EU country; a representative of the authority(ies) established for the EU institutions and bodies; a representative of the European Commission.’^110^ The Art 29 DPWP has delivered several opinions relating to data reuse. Thus far, notable opinions relate to consent^111^ and the roles of data controllers and processors.^112^

It is suggested here that establishing a dedicated Working Party, with a similar composition to Art 29 DPWP, but which is committed to considering purely those issues arising out of reuse of data for health related research, would offer significant benefits. First and foremost, such a group could deliver opinions which specifically respond to the challenges and concerns encountered by key actors who are reliant upon gaining access to health data for research purposes. Such opinions would also aid data custodians who are charged with the responsibility of determining when to share data. Under Article 30 of the EuDPD, Art 29 DPWP is charged with the following tasks:

(a) examine any question covering the application of the national measures adopted under this Directive in order to contribute to the uniform application of such measures;
(b) give the Commission an opinion on the level of protection in the Community and in third countries;
(c) advise the Commission on any proposed amendment of this Directive, on any additional or specific measures to safeguard the rights and freedoms of natural persons with regard to the processing of personal data and on any other proposed Community measures affecting such rights and freedoms;
(d) give an opinion on codes of conduct drawn up at Community level.^113^

It is proposed here that a new Data Reuse for Health Research Working Party would be charged with the above tasks as they relate to the new Data Protection Regulation should it be adopted. The Working Party could also be charged with clarifying whether pharmacoepidemiological studies are included in this sphere. Additional tasks of the Working Party would include: (a) drafting European level Guiding Principles of data reuse for health research, and (b) consulting on the potential for drafting new dedicated legislation governing health data reuse in research. Both of these tasks are considered next.

#### 5.1.1 Guiding Principles and Best Practice

Introducing a key set of guiding principles, supported by examples of best practice context could provide many benefits for researchers and data custodians alike. Principles should be seen as fundamental starting points to guide deliberation and action.^114^ Principle-based approaches in this context are ‘the use of broadly-stated objectives, standards and values by which individuals and institutions should conduct themselves when using data for research purposes.’^115^ The merits of principle-based approaches in the health research context have been discussed elsewhere.^116^ However, in order to help orient readers to the value which guiding principles could bring in this context, key points relating to adopting principle-based approaches to decision making are recounted here.

As we have considered, the complex legislative landscape is difficult to navigate. Overarching principles can offer clear articulations not only to guide action on everyday practices, but also to offer a set of standards against which conduct should be measured. This has been the approach adopted in the Scottish context of data reuse for health research by the Scottish Health Informatics Programme (SHIP).^117^ This Scotland-wide project which is dedicated to maximising the research potential of data reuse of electronic health records has adopted a set of Guiding Principles and Best Practice, subsequently endorsed by the Scottish Information Commissioner’s Office and adopted by the Scottish Government for its Data Linkage Framework.^118^

A set of Guiding Principles can provide decision-makers across the EU with ‘broad-based values and commonly-agreed objectives to determine through deliberation and reflection what action best fits in accordance with the particular value(s) advanced.^119^ Having a pre-determined, clear set of values can compensate for the gaps in the law where a clear course of action for the situation at hand is not offered. It is impossible to legislate on every perceivable eventuality; principles offer flexibility and guidance where provisions are not provided within the law.^120^ Principles can help to provide transparency to decision-makers and to data subjects alike, about the standards against which conduct will be measured.

The very process of drafting key principles and consulting key stakeholders across the EU can be of value in itself. This iterative process has the potential to uncover previously overlooked challenges to data reuse, which nonetheless require attention and clarification. A Principle-based approach could also tend to the current gaps which pharmacoepidemiological research falls into, it would promote sharing in appropriate contexts rather than forcing the research community to guess whether their activities (for example pharmacoepidemiological studies) and the associated data fit within certain provisions. Common principles can engender ‘mutual respect between all stakeholders and participants’.^121^

It is necessary also to acknowledge some weaknesses of principle-based approaches. Principles have often been criticised for being vague and abstract in nature,^122^ leaving significant scope for interpretation,^123^ and a lack of prescription can give rise to confusion about how to interpret and apply principles.^124^ As Knoppers et al. note when considering principles: ‘…discussion on the nature of such principles and their procedural translation in different contexts will necessarily vary.’ However it is argued here that the benefits of offering over-arching principles at the European level which have been mentioned above outweigh the limitations associated with principles.

#### 5.1.2 Consultation On New Legislation

The merits of new legislation are discussed in more detail below. Conducting a consultation on what this new legislation should and should not include could be one of the key tasks for the Working Party. It will be best placed, having drafted guiding principles and having dealt with queries from Member States (of the EU), to draft an informed legislative proposal for wider consultation.

### 5.2 New and Dedicated Legislation Governing Data Reuse

This next suggestion for reorienting the landscape moves one step further: introducing legislation specifically on the reuse of health data for research is proposed. As mentioned above, reuse of data for health research is proliferating, and will continue to do so as we move towards establishing new and strengthening pre-existing e-health infrastructures. Rather than relying upon building-in provisions for reuse of data within a larger regulation such as the proposed Data Protection Regulation, it is worthwhile considering introducing legislation which from the outset, is designed to legislate on health data reuse in the research context. It is argued here that having clear legislative articulations dedicated to the specific context of data reuse is a necessary starting point and cultural change is unlikely to occur without explicit reference to the need for sharing such data.

It has already been acknowledged above that revising, let alone drafting European-level legislation is by no means an easy feat, as exemplified by the duration of the drafting process for revisions to the current Directive. However, it is questionable whether this would be any more of an undertaking than the current ambitious task of revising the EuDPD as it stands. This proposal is for a clear and coherent Directive/Regulation which at its core, strives to enable data sharing activities to secure patient safety, and which acknowledges that this can occur in the context of wider pharmacoepidemiological research which necessitates reuse of patient data which does not only take place under pharmacovigilance activities.

Introducing new legislation is akin to taking a rule-based approach to regulation.^125^ This can offer more concreteness in comparison to principles and prescriptive^126^ articulations of conduct required from activities related to data reuse for research. In turn, dedicated legislation could provide a greater degree of certainty for researchers and custodians than the current legislative ‘patchwork’ which must be interpreted. As we considered above, the wide scope of the current Directive contains but a few mere exemptions which directly relate to the processing of data for health research purposes and lack clarity around key concepts such as pseudonymisation and personal data. Like principle-based approaches, rule-based approaches also have some shortcomings, most notably in their rigidity and the impossibility of anticipating and thus legislating for absolutely every possible eventuality.^127^

It is proposed here that a dedicated working party, new legislation, in tandem with a set of clear guiding principles might be considered as options to improve the status quo. Such a dual-approach would allow more prescriptive legislation and overarching principles to tend to the respective short-falls of each other. In particular, rules can offer clear and definitive guidance where possible, and principles can offer flexibility for those issues which require more (and inevitable) discretion on the part of decision-makers. As has been acknowledged earlier in this article, there are some cultural issues around sharing which legislation alone cannot overcome alone, however including clear articulations within legislation that enables and promotes important reuse of data for patient safety can act as a driver for cultural change in attitudes to and confidence around data sharing and use.

### 5.3 Openness of Data: Additional Consideration

The recently approved Clinical Trials Regulation^128^ attempts to encourage more transparency and openness of data. The new Regulation will require detailed and plain language summaries of clinical trial data to be posted on a database set up and run by the European Medicines Agency, these summaries and final trial reports will also be freely and publicly accessible on a central database. The major UK funding councils have recently begun explicitly to push the Open Access agenda whereby data and results must be available to the research community who may wish to verify research findings. This necessarily implicates data sharing and some funding is conditional upon these terms being fulfilled. It is worthwhile considering whether and if so, how European data protection legislation will progress towards promoting transparency and openness in the same way that the new Clinical Trials Regulation aspires to. It is also worth asking whether and if so, how the research community will be guided in terms of adhering to Open Access requirements whilst simultaneously negotiating with the current ill-fitting Directive. It also remains to be seen how the demands of a future Data Protection Regulation will sit alongside aspirations to create ‘a second open science revolution.’^129^

## 6 Conclusion

This article has highlighted the clear and pressing need for a shift in data protection legislation to explicitly recognise the value in and importance of data reuse in research. Neither the current EuDPD nor the proposed Albrecht amendments to the Draft Regulation adequately reflect this need. The important principle of promoting sharing is overshadowed by the need to protect privacy. Compromising privacy is by no means advocated or suggested by this author. It is clear that ‘data sharing is not in opposition to privacy and should be conducted in a responsible way such that it does not infringe on the privacy rights of individuals and groups’.^130^ The key message here is that there is a need to realign the current legislative regime in order to enable responsible reuse of health data for research.

One can sympathise with those charged with drafting the regulation, which must extend across the European Union and given the array of stake-holders and data-related activities which must be regulated. This formidable legislative undertaking does not however abrogate the fact that important activities may be hampered significantly, a concern reflected amongst the research community in their consideration of the proposed amendments.^131^ Pharmacoepidemiological studies may particularly suffer if proposals are adopted to consider pseudonymised data as ‘personal data’, to require consent, and where this is not possible, to require exceptionally high public interest thresholds must be satisfied. Due urgency is not being granted to facilitating pharmacoepidemiological studies and those studies relying on data reuse for health research more broadly. The legislative landscape must reorientate towards promoting and enabling reuse of health data for research.

We must acknowledge that the law can take significant steps in reshaping the regulatory landscape, but cultural attitudes and practices towards data reuse play an undeniable role and can be equally difficult to change.^132^ It is argued here that having clear legislative articulations dedicated to the specific context of data reuse is a necessary starting point and cultural change is unlikely to occur without explicit reference to the need for sharing data for reuse in health research. The key step to liberating the research community from the shadow of uncertainty around data sharing is introducing clear and coherent legislation which explicitly recognises the value of data linkage upfront and encourages ethically and legally robust data sharing. This legislation should be coupled with the establishment of a dedicated working party tasked with considering issues specific to the context of data reuse in health research and with establishing guiding principles, which at the very least, could provide timely, meaningful and practical signposts on the specific issues related to reuse of data for health research. At best, these solutions could help us get back on track to realising the potential which data reuse has for securing patient safety.
